# Coronary artery ectasia associated with IgG4-related disease: a case report and literature review

**DOI:** 10.1186/s12872-023-03369-7

**Published:** 2023-07-12

**Authors:** Muyun Tang, Zhiyu Zhang, Liang Wang, Hao Qian, Wei Wu, Zhenyu Liu, Zhujun Shen, Hua Chen, Zhiwei Guo, Ran Tian, Shuyang Zhang

**Affiliations:** 1 Department of Cardiology, Peking Union Medical College Hospital, Chinese Academy of Medical Sciences & Peking Union Medical College, 1 Shuaifuyuan, Dongcheng District, Beijing, China; 2 Department of Rheumatology and Clinical Immunology, Peking Union Medical College Hospital, Chinese Academy of Medical Sciences & Peking Union Medical College, 1 Shuaifuyuan, Dongcheng District, Beijing, China; 3 Department of Cardiology, Fuwai Hospital, National Center for Cardiovascular Diseases, Chinese Academy of Medical Sciences and Peking Union Medical College, Beijing, China

**Keywords:** Coronary artery Ectasia, IgG4-related disease, Acute coronary syndrome

## Abstract

**Background:**

Coronary artery ectasia is defined as a local or diffuse dilatation of the coronary artery more than 1.5 times the diameter of the adjacent normal segment. The etiology of coronary artery ectasia is diverse, and rarely complicated with immunoglobulin G4-related disease (IgG4-related disease). A limited number of cases have been reported, with insidious onset, slow progression but poor prognosis.

**Case presentation:**

we report a patient with coronary artery ectasia combined with IgG4-related disease. He has been diagnosed with IgG4-related disease 5 years after his first percutaneous coronary intervention (PCI). Despite routine treatment with steroids, he develops a large coronary aneurysm and eventually died.

**Conclusions:**

It is suggested that a thorough evaluation should be performed when coronary artery ectasia is diagnosed. The factors such as manifestations of coronary artery thickening, typical imaging features, other aortas involvement, increased serum IgG4 level, etc. should be considered for early diagnosis of key etiologies.

## Background

Coronary artery ectasia is a rare disease defined as a localized or diffuse dilation of the coronary artery lumen that exceeds the diameter of an adjacent portion (considered as a reference point) by more than 1.5 times. The incidence is 1.2–4.9% in the literature [1, 2]. The etiology of coronary artery ectasia is diverse, including atherosclerosis, which is the most common cause, and it is also associated with Kawasaki disease, infectious septic emboli, connective tissue disease, arteritis, etc. [1]. However, report of coronary artery ectasia complicated with immunoglobulin G4 (IgG4)-related disease is very rare. Immunoglobulin G4 (IgG4)-related disease is a rare immune-mediated fibro-inflammatory disease that has only been reported in recent years [3, 4].Only 5% of them have coronary artery involvement, but those patients tend to have a poor prognosis [5]. Here, we present a case of an elderly male with acute myocardial infarction caused by IgG4-related coronary artery ectasia. The patient is diagnosed with IgG4-related disease five years after the first onset of acute myocardial infarction. He has recurrent myocardial infarction caused by a huge coronary aneurysm after regular steroid therapy, which eventually led to his death.

## Case presentation

**Fig. 1:**
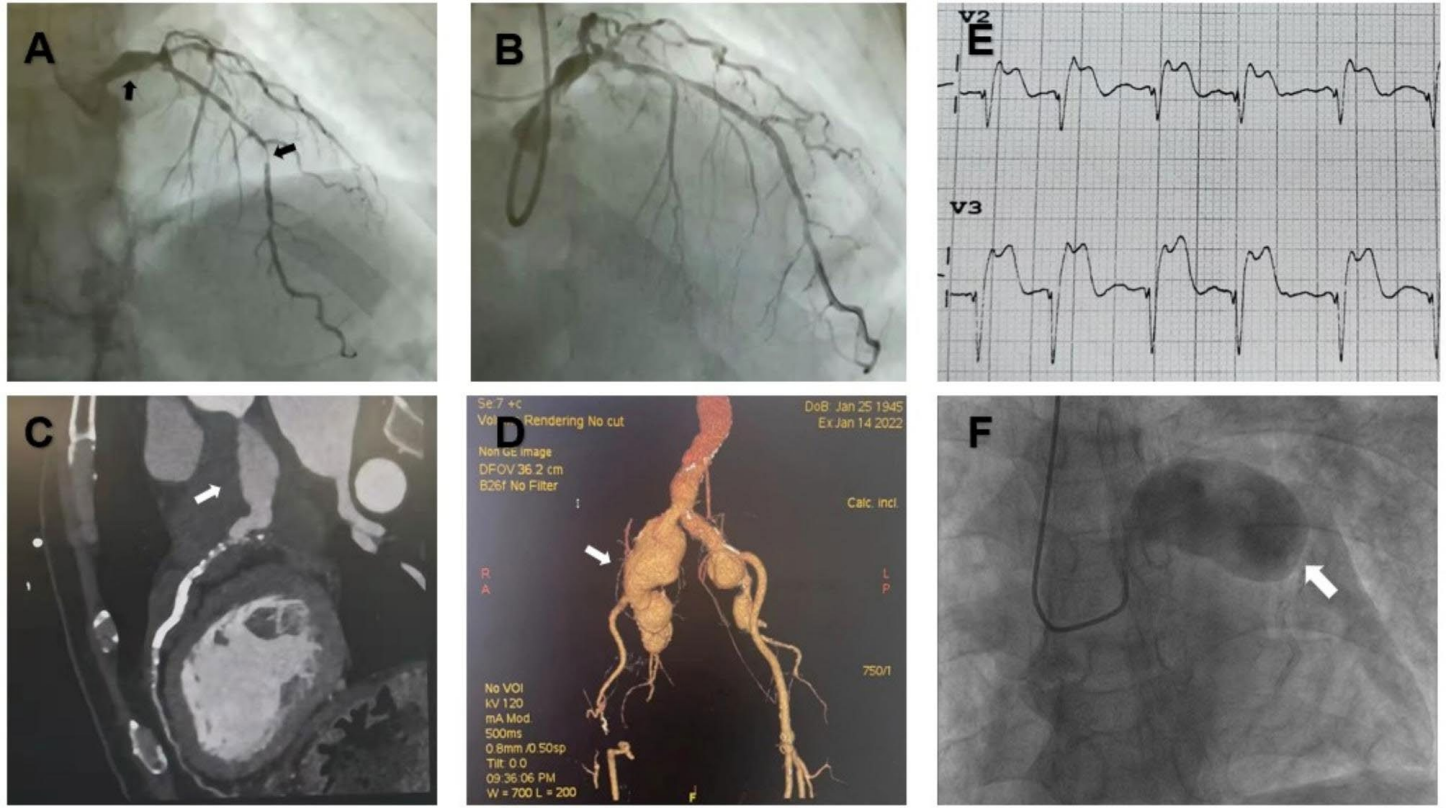
Imaging manifestations during the course of the patient’s disease. (A) Before stent implantation, LM ectasia and severe LAD stenosis (indicated by white arrows). (B) After stent implantation, LAD blood flow is restored. (C) Multiple calcifications in LAD and LM ectasia (indicated by white arrows). (D) Multiple aneurysms of the abdominal aorta and bilateral iliac arteries (indicated by white arrows). (E) Electrocardiogram of acute ST-segment elevation myocardial infarction. (F) A giant coronary artery aneurysm located in LM, about 28 mm in diameter (indicated by white arrows)

On September 2016, a 77-year-old male was diagnosed with non-ST-segment elevation myocardial infarction due to acute chest pain and underwent coronary angiography. Coronary angiography showed diffuse plaques in the left main coronary artery (LM), left anterior descending artery (LAD), left circumflex artery (LCX), and right coronary artery (RCA). There was an ectasia in LM with up to 40% limited terminal stenosis, and with stenosis up to 90% in the mid-distal segment of LAD, Therefore, the patient was implanted with a stent in the middle of the LAD (Fig. 1A,B), and then regularly took drugs for secondary prevention against coronary heart disease.

On October 2021, he had a recurrence of acute myocardial infarction. Computed tomography angiography (CTA) of the coronary artery and aorta showed that the wall of the proximal LM was significantly thickened, with a giant aneurysm. RCA, LAD, and LCX showed extensive calcification, plaque, and stenosis (Fig. 1C). In addition, there were multiple plaque ulcers and perforating ulcers in the aorta with local mural thrombus, multiple aneurysms, and plaque ulcers in both iliac arteries, and right superficial femoral artery occlusion (Fig. 1D). Blood tests revealed erythrocyte sedimentation rate (ESR) was 70 mm/h. Immunoglobulin G (IgG) level was 43 g/L, with an IgG4 level of 18.6 g/L. Antinuclear antibody (ANA) and anti-neutrophil cytoplasmic antibodies (ANCA) were negative. He was diagnosed with IgG4-related disease with multiple arterial involvements. However, due to significantly elevated inflammatory indicators of IgG4-RD at that time, he received conservative treatment with medications instead of intervention, Dual antiplatelet therapy, prednisone 40 mg/d and mycophenolate mofetil (MMF) 0.5 g bid were prescribed, and his chest pain was relieved. 1 month later, reexamination showed normal C-reactive protein (CRP) level and ESR level. Then prednisone was gradually reduced to 17.5 mg/d.

**Fig. 2:**
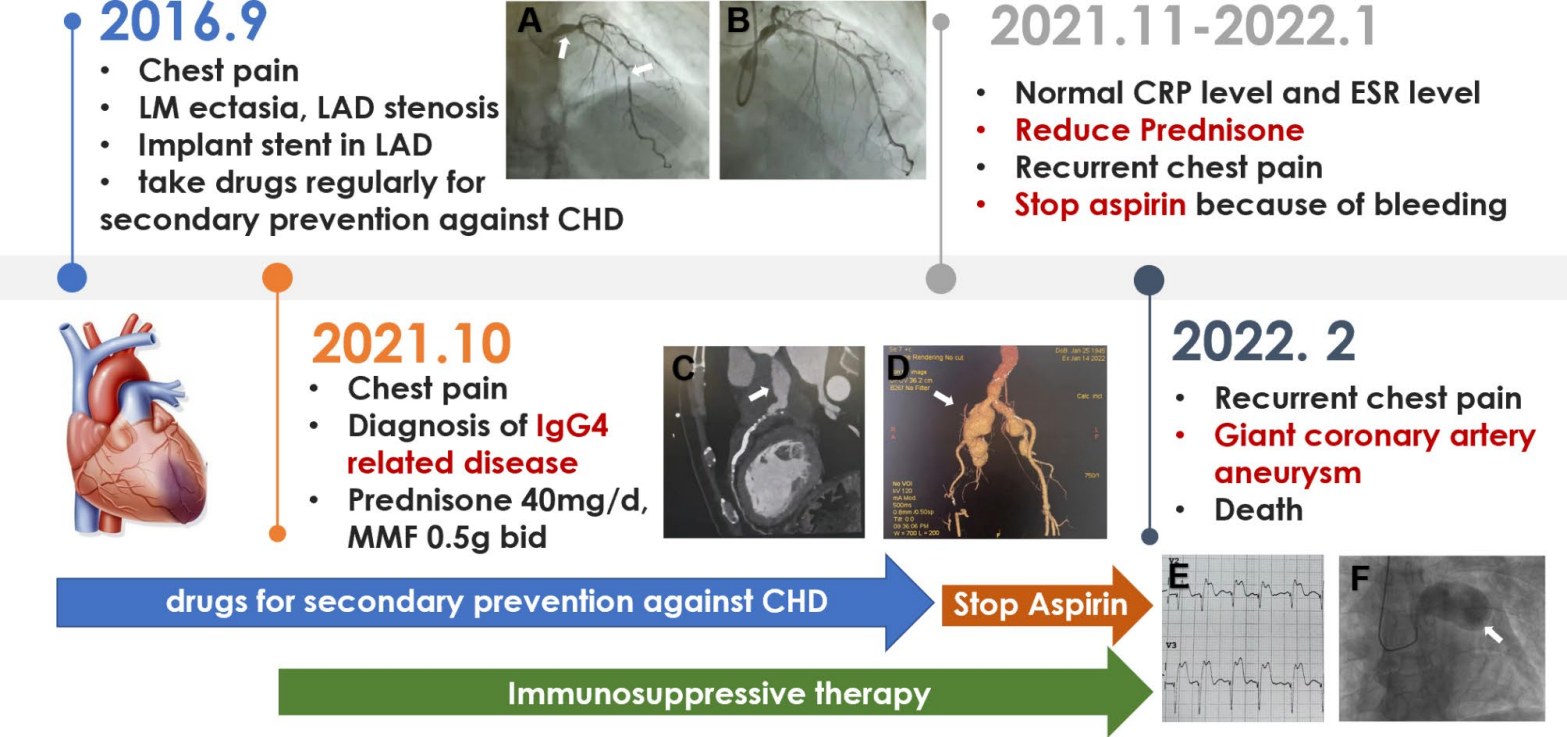
Time line

However, on January 2022, he suffered recurred chest pain again, and the symptoms were attributed to the recurrence of IgG4-related disease, thus prednisone was increased to 40 mg/d, but aspirin was discontinued because of multiple positive fecal occult blood test results and anemia. On February 3th, he had a sudden squeezing chest pain and was admitted to the emergency department. Electrocardiogram showed obvious ST-segment elevation in AVR and V2-V3 leads with ST-segment depression in II, III and AVF leads. Coronary angiography showed significant aneurysm-like ectasia of LM with a diameter of 28 mm (Figs. 1F), 90% stenosis and segmental ectasia in RCA, LAD and 100% occlusion in LCX and LAD. CRP level was increased to 59.5 mg/L. He recieved aspirin 100 mg qd, clopidogrel 75 mg qd, atorvastatin 20 mg qn, prednisone 40 mg qd, plus CTX 100 mg qod and relieved. The cardiac surgeon assessed that he had an indication for coronary artery bypass grafting (CABG), but his severe coronary and peripheral vascular lesions led to extremely high surgical risk and mortality, his family requested conservative medical treatment; on February 7, 2022, he suffered another sudden acute ST-segment elevation myocardial infarction involving the extensive anterior wall with atrial fibrillation and ventricular tachycardia, his family refused invasive operation, then he was treated with beta-blockers, lidocaine, epinephrine, and eventually died after being rescued ineffectively. Figure 2 shows the timeline of the patient’s entire course.

## Discussion and conclusions

The natural course of coronary artery ectasia is relatively unknown, [2] which makes early clinical management difficult. IgG4-related disease is also rare and has a wide variety of clinical manifestations, which is divided into four main phenotypes based on involved organs: Pancreato-Hepato-Biliary disease; Retroperitoneal Fibrosis and/or Aortitis; Head and Neck-Limited disease; Classic Mikulicz Syndrome with systemic involvement [6]. Studies have shown that IgG4-related disease involving the coronary arteries is usually accompanied by other aortic involvement [5]. In this case, multiple arteries are involved and a giant aneurysm ispresented in the coronary artery. Case reports of coronary aneurysms in combination with IgG4-related disease have been reviewed and listed in Table 1. It could be concluded that most of the patients are male and older than 60 years old. When the aneurysm is small or in the early stage of IgG4 disease, treatment with corticosteroids is usually responsive, but when a giant aneurysm is formed or IgG4 disease has progressed to an advanced stage, steroid treatment is not effective and surgery such as CABG might be a viable treatment. Furthermore, it is important to monitor aneurysms in patients with IgG4-related disease even under corticosteroid therapy. IgG4-related disease progresses insidiously and slowly, and is not easily diagnosed in the early stages. However, when the coronary arteries are involved, Risk of acute events such as heart attack may increase and the prognosis is poor.

**Table 1:** Literature Review of IgG4-Related Disease with Coronary Artery Aneurysms

Gender	IgG4-RD course		IgG4-RD manifestations	IgG4-RD treatment	Age of CAD onset	Clinical manifestations of CAD	Coronary aneurysm location and size	Coronary aneurysm treatment	Disease Outcome	Reference
Male	9 years		IgG level 2988 mg/dL ; RCAA 20 × 20 mm IgG4 level 155 mg/dL (when CAD onset)	Prednisolone 5 mg/d	74	Sudden chest pain; Elevated II and III on ECG;	RCAA 85 × 65 mm	CABG	Coronary artery aneurysm shrinks; Myocardial ischemia improves	Matsuyama et al., 2020 [19]
Male		Same as CAD	Asthma; colitis; Biopsy confirmed IgG4 colon involvement	Not mentioned	59	Chest pain; ST-segment depression	Multifocal aneurysm; ectasia and stenosis involving RCA, LAD and LCX	CABG	Good prognosis	Ansari-Gilani et al., 2020 [20]
Male		9 months	Plasma cell tumor; Right and left coronary artery aneurysms, maximum diameters of 40 and 25 mm, respectively IgG4 161 mg/dL	Antiplatelet and anticoagulant therapy; Prednisone	69	Coronary angiography	Aneurysm enlargement; Right (max 53 mm) and Left (max 40 mm) coronary artery aneurysms IgG4 level 306 mg/dL	CABG	Not mentioned	Bito et al., 2014 [21]
Male		9 years	Elevated serum IgG4 level; Right and Left coronary arteries, right internal iliac artery dilated; Extensive lymphocytic infiltration in CAA with IgG4/IgG ratio greater than 40%	MMF and Prednisolone	73	Tachypnea, sweating	LAD:5 cm; RCA:10 × 9 × 8 cm;	Cardiopulmonary Resuscitation	Death due to IgG4 syndrome with CAA coronary thrombosis	Chan, 2022 [22]
Male		Same as CAD	Kidney biopsy diagnosed as IgG4-related sclerosing disease	Corticosteroids	64	Dyspnea, weight loss, and fatigue	LCX: 11 × 9 cm; small right coronary aneurysm	Aspirin, and proton pump inhibitor therapy	relieved	Debonnaire et al., 2012 [23]
Male		12 years	IgG4 level 555 mg/dL (when CAD onset)	15 mg/d steroid	62	Annual follow-up CT;	LCX: maximum diameter of 38 mm	Off-pump CABG and Endovascular Hybrid Therapy	Discharged after 17 days without complications	Kamikawa et al., 2021 [24]
Male		Same as CAD	IgG4 level 1360 mg/ dL; Numerous IgG4-positive plasma cells with extensive fibrosis in axillary lymph node; Abdominal aortic and common iliac arteries involvement	35 mg/d prednisolone (0.6 mg/kg body weight) to 5 mg/d maintenance therapy	68	Dyspnea on exertion	Right coronary artery aneurysm	Not mentioned	Improvement in arterial wall thickening; Reduction in right coronary aneurysm; IgG4 level dropped to 238 mg/dL	Kan-o et al., 2015 [13]
Male		Same as CAD	Recurrent cerebral infarction, IgG4 1,350 mg/dL; Multiple RCA aneurysms with a maximal minor axis diameter of 11 mm; Left coronary artery diffusely dilated	Balloon angioplasty	60	2 years after balloon angioplasty. follow-up coronary angiography and CTA	proximal aneurysm still enlarged, distal aneurysm has formed an intraluminal thrombus; a new aneurysm developed	steroid therapy 20 mg/d, gradually reduced to 10 mg/d within six months	IgG4 level decreased to 187 mg/dL, aneurysm did not expand, RCA blood flow improved significantly to TIMI grade 3	Nishimura et al., 2016 [17]
Male		Same as CAD	IgG4 level 2607 mg/dL; History of allergic disease; Inflammatory cell infiltration in skin and lymph nodes	High-dose prednisolone: 1 mg/Kg per day	65	Subacute anterolateral ST-segment elevation myocardial infarction	Multifocal aneurysm, involving distal LM, LAD and LCX with large irregular thrombus (25 mm)	Antiplatelet and anticoagulant therapy	After 6 months, stable	Ruggio et al., 2018 [25]
Male		Same as CAD	Jaundice, dry cough, dry mouth; Elevated serum IgG4 levels; IgG4-expressing plasma cells in salivary glands, common bile duct, gall bladder wall, and pancreatic duct	Cyclophosphamide and corticosteroid	71	Echocardiography and coronary CTA	The right and left main trunks were moderately dilated, and there was a tumefaction (24 × 25 mm) on the side of the right atrium	Not mentioned	After two years follow-up, no significant changes in blood vessels	Takei et al., 2012 [14]
Male		9 years	Multiple arterial aneurysms; retroperitoneal fibrosis; high serum IgG level: 5470 mg/dL	Prednisolone; azathioprine	65	1) 4 months after diagnosis, follow-up CT showed RCAA; 2) Acute infero-lateral wall ST-elevation myocardial infarction; 3) Recurrent chest pain; LAD CAA ruptured; 4) 3 months after surgery, the size of the CAAs increased.	1) RCAA: 5.3 cm × 5.4 cm × 6.4 cm;	1) Right coronary aneurysm resection and CABG 2) Balloon angioplasty; Dual antiplatelet therapy for 12 months; 3) bovine pericardium repaired and CABG; 4) coil embolization, prednisolone 5 mg/d	6 months after coil embolization, mid LAD and 2nd OM CAA completely thrombosed but increased size: LAD: 2.5 cm × 2.5 to 2.9 cm × 3.2 cm LCX: 3.3 cm × 3.2 to 3.7 cm × 4.8 cm	Pota et al., 2021 [26]

**Abbreviations**: IgG4-RD: IgG4-Related Disease; CAD: Coronary Artery Disease; IgG: Immunoglobulin G; ECG: Electrocardiogram; CABG: coronary-artery-bypass-grafting; CAA: Coronary artery aneurysm; RCAA: right coronary artery aneurysm; LAD: Left anterior descending artery; RCA: Right coronary artery; LCX: Left circumflex artery; LM: left main coronary artery; OM: obtuse marginal; TIMI: Thrombolysis in myocardial infarction; CTA: Computed Tomography Angiography

In terms of pathophysiological mechanisms, the high level of serum IgG4 may be related to the involvement of coronary artery in patients with IgG4 -related diseases, which often affects the outer layer of the arterial wall and manifest as thickened fibrosis lesions accompanied by IgG4-positive plasma cell infiltration [7, 8]. Besides, IgG4 may promote the development of low-density plaques through an immune-inflammatory response [9]. Intimal thickening due to pericoronitis may also cause physical compression, leading to coronary artery lumen narrowing[10]and subsequent coronary ectasia. According to the results of imaging, the lesions can be divided into types of stenotic, aneurysmal, and diffuse wall thickening [11]. Some large aneurysms can also show typical characteristics of the pigs-in-a-blanket sign [5, 8].

In terms of treatment, systemic corticosteroid therapy is the first-line treatment for IgG4-related diseases, and alternative immunosuppressive agents can also be used for maintenance and adjuvant therapy; In addition, as an emerging therapy, the therapy of targeting B and T lymphocyte activation remains to be further studied [12]. However, although studies had shown that steroid therapy could improve the wall thickening of IgG4-related aneurysms, high-dose steroids might cause a thinner and more fragile aneurysm wall, increasing the risk of rupture [11, 13, 14]. Therefore, individualized steroid therapy of an appropriate dose is critical. Moreover, as seen in Table 1, patients who are treated with steroid therapy only at advanced stages of the disease tended to have a poor prognosis, and this phenomenon had also been confirmed by related studies, which has found that inflammatory aneurysms are reversible in the early stage, but after the completion of vascular remodeling, the aneurysm had little response to corticosteroids [15]. In this case, the patient is diagnosed with IgG4-related disease and started steroid therapy 5 years after coronary artery ectasia is discovered, which might be responsible for the rapid progression of coronary aneurysm-like ectasia and eventually myocardial infarction, and atherosclerosis also appears to be a risk factor for poor prognosis [16]. In addition, since balloon angioplasty might lead to the formation of new aneurysms, and stent implantation could also present stent migration and occlusion caused by the progression of a coronary artery aneurysm, PCI and balloon angioplasty should be carefully considered and more attention as well as monitoring are necessary after intervention [17, 18]. In this patient, the existing LM coronary ectasia does not recover but worsened after systemic steroid treatment, which may be related to late treatment, steroid dose reduction, atherosclerosis, PCI, discontinuation of antiplatelet drugs, etc. (Fig. 2)

In conclusion, this study reports a rare case of IgG4 combined with coronary artery ectasia in which the patient’s disease rapidly progresses to form a giant aneurysm, leading to recurrent acute infarction and eventual death, possibly due to delayed hormone therapy, decreased hormone dosage, and discontinuation of aspirin, etc. This suggests that screening of coronary arteries may need to be emphasized in IgG4-RD, as well as the importance of early intervention. On the other hand, for atypical coronary lesions such as coronary ectasia, we should consider other etiologies in addition to atherosclerotic disease, including rare IgG4-related disease. If there is coronary thickening, typical imaging features, other aortic involvement and inflammation indicators like elevated serum IgG4, a high degree of vigilance is required to diagnose IgG4-related disease and start anti-inflammatory immunosuppressive therapy at an early stage. Also, we should be cautious in progress of PCI and balloon angioplasty, focus on monitoring and be alert for thrombosis. If necessary, surgical intervention can be performed in some patients.

## Data Availability

The datasets used in the case are available from the corresponding author upon reasonable request.

## Abbreviations

IgG4-RD: Immunoglobulin G4-related disease
PCI: Percutaneous coronary intervention
CAE: Coronary artery ectasia
LM: Left main coronary artery
LAD: Left anterior descending artery
LCX: Left circumflex artery
RCA: Right coronary artery
CRP: C-reactive protein
ESR: Erythrocyte sedimentation rate
IgG: Immunoglobulin G
ANCA: Anti-neutrophil cytoplasmic antibodies
MMF: Mycophenolate mofetil
CABG: Coronary artery bypass grafting
CAD: Coronary Artery Disease
ECG: Electrocardiogram
CAA: Coronary artery aneurysm
RCAA: right coronary artery aneurysm
OM: obtuse marginal
TIMI: Thrombolysis in myocardial infarction
CTA: Computed tomography angiography
